# Triglyceride glucose index is a useful marker for predicting subclinical coronary artery disease in the absence of traditional risk factors

**DOI:** 10.1186/s12944-020-1187-0

**Published:** 2020-01-14

**Authors:** Gyung-Min Park, Young-Rak Cho, Ki-Bum Won, Yu Jin Yang, Sangwoo Park, Soe Hee Ann, Yong-Giun Kim, Eun Ji Park, Shin-Jae Kim, Sang-Gon Lee, Dong Hyun Yang, Joon-Won Kang, Tae-Hwan Lim, Hong-Kyu Kim, Jaewon Choe, Seung-Whan Lee, Young-Hak Kim

**Affiliations:** 1 Division of Cardiology, Ulsan University Hospital, University of Ulsan College of Medicine, 877 Bangeojinsunhwando-ro, Dong-gu, Ulsan, 44033 Republic of Korea; 2 Division of Cardiology, Dong-A University Hospital, Busan, Republic of Korea; 3 Division of Cardiology, Asan Medical Center, University of Ulsan College of Medicine, Seoul, Republic of Korea; 4 Medical information Center, Ulsan University Hospital, Ulsan, Republic of Korea; 5 Division of Radiology, Asan Medical Center, University of Ulsan College of Medicine, Seoul, Republic of Korea; 6 Division of Health Screening and Promotion Center, Asan Medical Center, University of Ulsan College of Medicine, Seoul, Republic of Korea

**Keywords:** Triglyceride glucose index, Atherosclerosis, Risk factor, Coronary computed tomographic angiography

## Abstract

**Background:**

Atherosclerotic cardiovascular (CV) events commonly occur in individuals with a low CV risk burden. This study evaluated the ability of the triglyceride glucose (TyG) index to predict subclinical coronary artery disease (CAD) in asymptomatic subjects without traditional CV risk factors (CVRFs).

**Methods:**

This retrospective, cross-sectional, and observational study evaluated the association of TyG index with CAD in 1250 (52.8 ± 6.5 years, 46.9% male) asymptomatic individuals without traditional CVRFs (defined as systolic/diastolic blood pressure ≥ 140/90 mmHg; fasting glucose ≥126 mg/dL; total cholesterol ≥240 mg/dL; low-density lipoprotein cholesterol ≥160 mg/dL; high-density lipoprotein cholesterol < 40 mg/dL; body mass index ≥25.0 kg/m^2^; current smoking; and previous medical history of hypertension, diabetes, or dyslipidemia). CAD was defined as the presence of any coronary plaque on coronary computed tomographic angiography. The participants were divided into three groups based on TyG index tertiles.

**Results:**

The prevalence of CAD increased with elevating TyG index tertiles (group I: 14.8% vs. group II: 19.3% vs. group III: 27.6%; *P* < 0.001). Multivariate logistic regression models showed that TyG index was associated with an increased risk of CAD (odds ratio [OR] 1.473, 95% confidence interval [CI] 1.026–2.166); especially non-calcified (OR 1.581, 95% CI 1.002–2.493) and mixed plaques (OR 2.419, 95% CI 1.051–5.569) (all *P* < 0.05). The optimal TyG index cut-off for predicting CAD was 8.44 (sensitivity 47.9%; specificity 68.5%; area under the curve 0.600; *P* < 0.001). The predictive value of this cut-off improved after considering the non-modifiable factors of old age and male sex.

**Conclusions:**

TyG index is an independent marker for predicting subclinical CAD in individuals conventionally considered healthy.

## Background

Cardiovascular (CV) risk is clinically stratified based on traditional CV risk factors (CVRFs) in asymptomatic individuals [1]. However, although most adverse CV events are closely related to traditional CVRFs, atherosclerotic CV events commonly occur in individuals with a low CV risk burden [2–4]. According to current recommendations for primary prevention [5], asymptomatic individuals without CVRFs are usually not considered a target for preventive strategies, regardless of the potential presence of atherosclerosis. Recently, the Progression of Early Subclinical Atherosclerosis (PESA) study reported the noticeable results that subclinical atherosclerosis was observed in about 50% of the CVRF-free middle-aged population [6]. Despite some limitation of PESA study in that obesity was not included in CVRFs, this finding highlights the significance of identifying independent predictors for subclinical atherosclerosis in the absence of traditional CVRFs considering that subclinical atherosclerosis underlies most CV events. Although recent study has focused on the significance of low-density lipoprotein cholesterol (LDL-C) for subclinical coronary artery disease (CAD) in asymptomatic adults without CVRFs [7], data on the independent predictor for subclinical atherosclerosis in this population has been scarce in clinical practice.

Insulin resistance (IR) has been demonstrated to play an important role in CAD [8, 9]. The triglyceride glucose (TyG) index, which is calculated from fasting measurements of triglycerides and glucose, has been recently suggested as a reliable surrogate marker of IR [10–12]. Several previous studies have shown that TyG index is associated with coronary atherosclerosis [13, 14]. However, whether TyG index is a useful predictor of coronary atherosclerosis in healthy individuals with a low CV risk burden has not been determined. In the present study, we examined the association between TyG index and subclinical CAD in individuals without traditional CVRFs.

## Methods

### Study design and population

In this retrospective, cross-sectional, and observational study, a total of 8945 Korean individuals aged ≥40 years who had undergone self-referred coronary computed tomography angiography (CCTA) evaluation as part of a general health examination were consecutively enrolled at the Health Screening and Promotion Center of the Asan Medical Center between January 2007 and December 2011 [15]. Among these, 2023 individuals refused to participate in this study. Subsequently, 4972 were gradually excluded because of (a) a history of angina or myocardial infarction (*n* = 336); (b) abnormal resting electrocardiography results, including pathological Q waves, ischemic ST segments, T wave changes, or left bundle-branch blocks (*n* = 205); (c) structural heart disease (*n* = 49); (d) a history of open heart surgery (*n* = 5); (e) a history of percutaneous coronary intervention (*n* = 5); (f) a previous cardiac procedure (*n* = 10); (g) renal insufficiency, defined as serum creatinine ≥0.13 mmol/L (≥1.5 mg/dL) (*n* = 1); (h) traditional CVRFs, defined as fasting glucose ≥126 mg/dL, hemoglobin A1C (HbA1C) levels ≥6.5%, or previous medical history of diabetes (*n* = 1105); systolic blood pressure ≥ 140 mmHg, diastolic blood pressure ≥ 90 mmHg, or previous medical history of hypertension (*n* = 1727); total cholesterol ≥240 mg/dL, LDL-C ≥ 160 mg/dL, high-density lipoprotein cholesterol (HDL-C) < 40 mg/dL, or previous medical history of dyslipidemia (*n* = 1199); body mass index (BMI) ≥25.0 kg/m^2^ (*n* = 659); and current smoking (*n* = 290); or (i) incomplete laboratory data (*n* = 81). Finally, 1250 asymptomatic individuals were enrolled in the present study (Additional file 1: Figure S1). The study protocol was approved by the local ethics committee of Asan Medical Center. All patients provided written informed consent.

Basic demographic data were acquired from a database maintained by the Health Screening and Promotion Center of Asan Medical Center. All medical history was collected using a systematic questionnaire conducted before a general health examination. Height and weight measurements were obtained while the participants wore light clothing without shoes. BMI was calculated as weight in kilograms divided by the square of the height in meters. Obesity was defined as a BMI of ≥25.0 kg/m^2^, based on the cut-offs for Asian populations. Blood pressure was measured using an automatic manometer with an appropriate cuff size after the participants rested for ≥5 min. Blood samples were drawn from the antecubital vein into vacuum tubes after the participants fasted overnight and were analyzed at the certified central laboratory of Asan Medical Center. Total cholesterol, triglyceride, LDL, HDL, uric acid, and creatinine were measured by the enzymatic colorimetric method using a Toshiba 200FR Neo (Toshiba Medical System Co., Ltd., Tokyo, Japan). Fasting glucose was measured by the enzymatic colorimetric method using a Toshiba 200 FR auto-analyzer (Toshiba). Ion-exchange high-performance liquid chromatography (Bio-Rad Laboratories, Inc., Hercules, CA) was performed for the measurement of HbA1C. The TyG index was calculated as ln [fasting triglycerides (mg/dL) × fasting glucose (mg/dL) / 2]. Family history of CAD was defined as CAD in a first-degree relative of any age. The Framingham risk score was calculated for all participants [16–18].

### Acquisition and analysis of CCTA images

CCTA was performed using a dual-source CT scanner (Somatom Definition; Siemens, Erlangen, Germany) or a single-source 64-slice CT scanner (LightSpeed VCT; GE, Milwaukee, WI). Patients without contraindications to beta-adrenergic blocking agents and with initial heart rates > 65 bpm received an oral dose of 2.5-mg bisoprolol (Concor, Merck, Darmstadt, Germany) 1 h before the CT examination. CT scanning was performed in the retrospective ECG-gating mode with ECG-based tube current modulation or the prospective ECG-triggering mode. Before contrast injection, isosorbide dinitrate (Isoket spray; Schwarz Pharma, Monheim, Germany) was used. Following this, 60–80 mL of iodinated contrast (Iomeron 400; Bracco, Milan, Italy) was injected at 4 mL/s, followed by a 40-mL saline flush during CCTA acquisition. The region of interest was placed in the ascending aorta, and image acquisition was automatically initiated once a selected threshold (100 HU) had been reached using bolus tracking. A standard scanning protocol was used and the tube voltage and tube current-time product were adjusted according to the patient’s body size as follows: 100 or 120 kVp tube voltage; 240–400 mA per rotation (dual-source CT), and 400–800 mA (64-slice CT) tube current. The size-specific dose estimate was calculated using the patient’s body diameter [19]. The mean effective dose for our CT protocol was 7.6 ± 5.1 mSv. All CCTA images were analyzed using a dedicated workstation (Advantage Workstation, GE; or Volume Wizard, Siemens) by experienced cardiovascular radiologists (D.H.Y., J.-W.K., and T.-H.L.) according to the Society of Cardiovascular Computed Tomography’s guidelines [20]. The coronary artery calcium score (CACS) was determined as previously described [21]. CACS was categorized into five groups: 0, 1–10, 11–100, 101–400, and > 400. Plaques were defined as structures ≥1 mm^2^ within and/or adjacent to the vessel lumen, which were clearly distinguishable from the lumen and the surrounding pericardial tissue. Plaques without calcium were classified as non-calcified, those with calcified tissue comprising ≥50% of the plaque area (density > 130 HU) were classified as calcified, and those with < 50% calcium were classified as mixed plaques [22]. The contrast-enhanced portion of the lumen was semi-automatically traced at the site of maximal stenosis and compared with the mean value of the proximal and distal reference sites [23]. CAD was defined as the presence of any coronary plaque.

### Statistical analysis

Continuous variables are expressed as mean ± SD. Categorical variables are presented as absolute values and proportions. Continuous variables were compared using one-way analysis of variance. Categorical variables were compared using the χ^2^-test or Fisher’s exact test, as appropriate. Univariate regression analysis was performed to evaluate the association between clinical variables and CAD. Variables with *P* < 0.05 on univariate analysis were considered as confounding factors and entered the multivariate regression analysis. ROC curve analysis using the Youden index was conducted to determine the optimal TyG index cut-off for predicting CAD. All statistical analyses were performed using the Statistical Package for the Social Sciences version 19 (SPSS, Chicago, Il) and SAS version 9.1.3 (SAS Institute Inc., Cary, NC). A *P*-value of < 0.05 was considered significant for all analyses.

## Results

### Baseline characteristics

Table 1 shows the clinical characteristics of the study cohort. The mean age of the 1250 participants (586 men, 46.9%) was 52.8 ± 6.5 years; 75 (6%) participants were older than 65 years. All participants were divided into three groups based on TyG index tertiles. The mean TyG indices were 7.84 ± 0.19, 8.27 ± 0.11, and 8.83 ± 0.30 in groups I (lowest), II, and III (highest), respectively. There was no significant difference in age among the three groups. Systolic and diastolic blood pressure, BMI, Framingham risk score, the levels of total cholesterol, triglyceride, LDL-C, creatinine, fasting glucose, HbA1C, and uric acid, and the prevalence of male sex significantly increased with increasing tertiles. The levels of HDL-C significantly decreased with increasing tertiles. The overall prevalence of CAD was 20.6%. Compared with participants < 65 years, those ≥65 years had a significantly higher prevalence of CAD (18.5% vs. 53.3%, *P* < 0.001) (Additional file 2: Figure S2A). Men had a higher prevalence of CAD than women (33.8% vs. 8.9%, *P* < 0.001) (Additional file 2: Figure S2B). The prevalence of categorical CACS was significantly different among the three groups. The prevalence of any, calcified, non-calcified, and mixed plaque significantly increased with increasing tertiles.

**Table 1:** Clinical characteristics

	Total (*n* = 1250)	Tertile of TyG index			
		I (lowest) (*n* = 413) 6.98–8.08	II (*n* = 424) 8.09–8.47	III (highest) (*n* = 413) 8.48–10.17	*P*
Age, years	52.8 ± 6.5	52.3 ± 6.4	52.7 ± 6.6	53.2 ± 6.5	0.133
Age ≥ 65 years, n (%)	75 (6)	20 (4.8)	26 (6.1)	29 (7.9)	0.415
Male, n (%)	586 (46.9)	156 (37.8)	181 (42.7)	249 (60.3)	< 0.001
Systolic BP, mmHg	113.2 ± 10.7	111.6 ± 10.8	112.5 ± 10.4	115.5 ± 10.4	< 0.001
Diastolic BP, mmHg	71.3 ± 8.8	69.9 ± 8.9	70.5 ± 8.9	73.6 ± 8.2	< 0.001
BMI, kg/m^2^	22.2 ± 1.7	21.7 ± 1.9	22.2 ± 1.6	22.8 ± 1.5	< 0.001
Family history of CAD, n (%)	196 (15.7)	53 (12.8)	63 (14.9)	80 (19.4)	0.030
Framingham risk score	3.9 ± 2.9	2.8 ± 2.4	3.5 ± 2.5	5.3 ± 3.2	< 0.001
Total cholesterol, mg/dL	192.3 ± 25.0	185.5 ± 26.7	193.0 ± 24.2	198.4 ± 22.4	< 0.001
Triglyceride, mg/dL	94.3 ± 48.6	55.9 ± 9.7	82.1 ± 10.8	145.2 ± 52.1	< 0.001
LDL-C, mg/dL	116.1 ± 22.2	107.9 ± 22.3	117.5 ± 21.0	122.9 ± 20.6	< 0.001
HDL-C, mg/dL	60.9 ± 13.2	66.9 ± 13.3	61.7 ± 12.0	54.1 ± 10.9	< 0.001
Creatinine, mg/dL	0.8 ± 0.2	0.8 ± 0.2	0.8 ± 0.2	0.9 ± 0.2	< 0.001
Fasting glucose, mg/dL	96.0 ± 8.8	92.4 ± 7.9	96.2 ± 8.3	99.5 ± 8.9	< 0.001
HbA1C, %	5.4 ± 0.4	5.4 ± 0.4	5.4 ± 0.4	5.5 ± 0.4	< 0.001
Uric acid, mg/dL	4.9 ± 1.2	4.6 ± 1.2	4.9 ± 1.2	5.3 ± 1.2	< 0.001
Categorical CACS, n (%)					0.001
0	1061 (84.9)	370 (89.6)	366 (86.3)	325 (78.7)	
1–10	69 (5.5)	16 (3.9)	23 (5.4)	30 (7.3)	
11–100	90 (7.2)	18 (4.4)	29 (6.8)	43 (10.4)	
101–400	25 (2.0)	6 (1.5)	5 (1.2)	14 (3.4)	
> 400	5 (0.4)	3 (0.7)	1 (0.2)	1 (0.2)	
Any plaque, n (%)	257 (20.6)	61 (14.8)	82 (19.3)	114 (27.6)	< 0.001
Calcified plaque, n (%)	157 (12.6)	38 (9.2)	50 (11.8)	69 (16.7)	0.004
Non-calcified plaque, n (%)	120 (9.6)	27 (6.5)	35 (8.3)	58 (14.0)	0.001
Mixed plaque, n (%)	32 (2.6)	5 (1.2)	9 (2.1)	18 (4.4)	0.013

Values are given as the mean ± standard deviation or number (%)

*P*-value of < 0.05 was considered significant

*BMI* Body mass index, *BP* Blood pressure, *CACS* Coronary artery calcium score, *CAD* Coronary artery disease, *HbA1C* Hemoglobin A1C, *HDL-C* High-density lipoprotein cholesterol, *LDL-C* Low-density lipoprotein cholesterol, *TyG* Triglyceride glucose

### Association between clinical variables and subclinical CAD

Univariate regression analysis showed that age; male sex; systolic and diastolic blood pressure; BMI; and the levels of LCL-C, HDL-C, and uric acid were significantly associated with the risk of CAD. Compared with that in group I, the risk of CAD was significantly higher in group III (OR 2.200, 95% CI 1.555–3.113, *P* < 0.001) (Table 2).

**Table 2:** Association between clinical variables and subclinical CAD

Variables	Univariate	
	OR (95% CI)	*P*
Age, 1 years increase	1.116 (1.092–1.141)	< 0.001
Male	5.233 (3.810–7.188)	< 0.001
Systolic BP, per 1 mmHg increase	1.051 (1.037–1.065)	< 0.001
Diastolic BP, per 1 mmHg increase	1.060 (1.043–1.078)	< 0.001
BMI, 1 kg/m^2^ increase	1.221 (1.121–1.331)	< 0.001
Family history of CAD	1.268 (0.884–1.819)	0.198
TyG index tertile		
I (lowest)	1	–
II	1.384 (0.962–1.990)	0.080
III (highest)	2.200 (1.555–3.113)	< 0.001
LCL-C, per 1 mg/dL increase	1.010 (1.003–1.016)	0.004
HDL-C, per 1 mg/dL increase	0.978 (0.967–0.988)	< 0.001
HbA1C, per 1% increase	1.379 (0.942–2.018)	0.098
Uric acid, per 1 mg/dL increase	1.567 (1.397–1.758)	< 0.001

*P*-value of < 0.05 was considered significant

*BMI* Body mass index, *BP* Blood pressure, *CAD* Coronary artery disease, *CI* Confidence interval, *HbA1C* Haemoglobin A1C, *HDL-C* High-density lipoprotein cholesterol, *LDL-C* Low-density lipoprotein cholesterol, *OR* Odds ratio

### Impact of TyG index on coronary plaques

Univariate regression analysis showed that TyG index was associated with an increased risk of CAD (OR 2.158, 95% CI 1.605–2.903, *P* < 0.001). In multivariate regression analysis, TyG index had an incremental impact on CAD (OR 1.473, 95% CI 1.026–2.166, *P* = 0.036). Regarding coronary plaque subtypes, multivariate regression analysis showed that TyG index was independently associated with non-calcified (OR 1.581, 95% CI 1.002–2.493, *P* = 0.049) and mixed plaque (OR 2.419, 95% CI 1.051–5.569, *P* = 0.038) (Table 3).

**Table 3:** Impact of TyG index on coronary plaques

Variables	OR (95% CI)	*P*
Any plaque		
TyG index, per 1 increase		
Model 1	2.158 (1.605–2.903)	< 0.001
Model 2	1.473 (1.026–2.166)	0.036
Calcified plaque		
TyG index, per 1 increase		
Model 1	2.016 (1.416–2.870)	< 0.001
Model 2	1.488 (0.965–2.295)	0.072
Non-calcified plaque		
TyG index, per 1 increase		
Model 1	2.294 (1.549–3.398)	< 0.001
Model 2	1.581 (1.002–2.493)	0.049
Mixed plaque		
TyG index, per 1 increase		
Model 1	3.012 (1.499–6.053)	0.002
Model 2	2.419 (1.051–5.569)	0.038

*P*-value of < 0.05 was considered significant

*BMI* Body mass index, *BP* Blood pressure, *CI* Confidence interval, *OR* Odds ratio, *TyG* Triglyceride glucose

Model 1: Unadjusted

Model 2: Adjusted for age, male sex, systolic and diastolic BP, BMI, and the levels of LDL-C, HDL-C, and uric acid

### Optimal TyG index cut-off for predicting subclinical CAD

The ROC analysis showed that the optimal TyG index cut-off for predicting subclinical CAD, as determined using the Youden index, is 8.44 (sensitivity: 47.9%; specificity: 68.5%; AUC: 0.600; 95% CI: 0.561–640; *P* < 0.001) (Additional file 3: Figure S3). The predictive power of TyG levels greater than 8.44 for CAD significantly improved after taking into account age ≥ 65 years (TyG > 8.44 vs. TyG > 8.44 mg/dL with age ≥ 65 years; AUC: 0.580 vs. 0.632; *P* < 0.001) and both age ≥ 65 years and male sex (TyG > 8.44 vs. TyG > 8.44 with age ≥ 65 years and male sex; AUC: 0.580 vs. 0.745; *P* < 0.001) (Table 4).

**Table 4:** Comparison of ROC models related to the cut-offs of TyG index for predicting subclinical CAD

ROC Models	AUC (95% CI)
TyG index ≥8.44	0.580 (0.546–0.614)
TyG index ≥8.44 with age ≥ 65 years	0.632 (0.596–0.667)*
TyG index ≥8.44 with age ≥ 65 years and male sex	0.745 (0.714–0.776)*

*P*-value of < 0.05 was considered significant

*AUC* Area under the curve, *CAD* Coronary artery disease, *CI* Confidence interval, *LDL-C* Low-density lipoprotein cholesterol, *ROC* Receiver operating characteristic

*P < 0.001 vs. TyG index ≥8.44

## Discussion

Individuals without traditional CVRFs have been considered relatively healthy and at low risk of developing atherosclerosis. Recently, PESA study reported that the prevalence of subclinical atherosclerosis in 4184 individuals without conventional CVRFs was 49.7%; however, this study had limitation in that obesity was not considered as traditional CVRFs. In the present study, we identified that the overall prevalence of CAD in asymptomatic CVRF-free Korean population was approximately 21% using same CVRFs criteria of PESA after including obesity in the CVRFs definition. The prevalence in those younger than 65 years was 18.5%, but the rate was approximately three-times higher in individuals ≥65 years. In addition, men had a significantly higher prevalence of CAD than women (33.8% vs. 8.9%). We were able to confirm that TyG index has significant value in predicting subclinical CAD in individuals without CVRFs. In terms of specific coronary plaque subtypes, TyG index was independently associated with an increased risk of non-calcified or mixed coronary plaques in this population.

It is important to identify independent predictors of subclinical CAD in individuals with a low CV risk burden considering the frequency of major atherosclerotic CV events in this population. Moreover, recent data showing that non-obstructive coronary plaques contribute to an increase in adverse CV events also suggest the significance of identifying predictors for subclinical CAD [24–26]. Previous studies had reported that TyG index was a reliable marker of IR and was associated with coronary artery calcification in a general population. However, the value of TyG index in detecting subclinical CAD in the absence of traditional CVRFs has not been evaluated. One interesting finding of the present study is that TyG index was independently associated with the risk of non-calcified plaques in the absence of CVRFs. Based on the results of a recent meta-analysis [27], non-calcified plaque could be associated with an increased risk of acute coronary syndrome events. Therefore, this finding supports the hypothesis that TyG index is an important CV risk factor independent of traditional CVRFs.

Compared with the results of PESA study, the prevalence of subclinical atherosclerosis was relatively low in the present study. This discrepancy might be related to the differences in (1) the method of measuring subclinical atherosclerosis, (2) the definition of CVRFs, and (3) the ethnicity of the participants. As is well known, obesity is strongly associated with atherosclerosis even in metabolically healthy population [28]. Considering that all the participants in our study were Korean, we excluded individuals with a BMI ≥25.0 kg/m^2^, which is the cut-off for the definition of obesity in Asian populations. Thus, it is possible that the prevalence of subclinical atherosclerosis was overestimated in the PESA study.

The present study identified the optimal cut-off of TyG index for predicting CAD in asymptomatic CVRF-free individuals. However, the AUC of 8.44 might be poor, suggesting that it is difficult to predict the presence of subclinical CAD based on TyG index alone. Although the predictive value improved after considering non-modifiable clinical factors, including old age and male sex, it remained only moderately powerful. However, this may be meaningful on a population level. Although data on the association between TyG index levels and adverse CV events in individuals with a low risk burden are limited, our result implies that it is important to keep the TyG index below the optimal cut-off in this population.

Some limitations were present in our study. First, all participants voluntarily visited the hospitals and participated in the general health examination. Thus, selection bias may be present. Second, the results may not be generalizable to other populations because all participants were Korean. Finally, the routine use of CCTA examinations in asymptomatic individuals remains unjustified, despite advances that have addressed its shortcomings [29]. Despite these limitations, the present study could provide a clinical implication in that we identified the independent predictive value of TyG index for subclinical CAD in the absence of CVRFs, especially in Asian population.

## Conclusions

In summary, TyG index is an independent marker of the presence of CAD, especially non-calcified or mixed plaques, in asymptomatic individuals without traditional CVRFs. Further large-scale prospective studies might be necessary to confirm the significance of TyG index in primary prevention of CAD in individuals who have conventionally been considered healthy.

## Supplementary information

**Additional file 1: Figure S1.** Overview of the study population.

**Additional file 2: Figure S2.** Prevalence of subclinical CAD according to old age and sex.

**Additional file 3: Figure S3.** Optimal TyG cut-offs for predicting subclinical CAD.

## Data Availability

The datasets used and analyzed during the current study are available from the corresponding author on reasonable request.

## Abbreviations

BMI: Body mass index
CACS: Coronary artery calcium score
CAD: Coronary artery disease
CCTA: Coronary computed tomography angiography
CI: Confidence interval
CV: Cardiovascular
CVRFs: Cardiovascular risk factors
HbA1C: Hemoglobin A1C
HDL-C: High-density lipoprotein cholesterol
LCL-C: Low-density lipoprotein cholesterol
OR: Odds ratio
ROC: Receiver operating characteristic
TyG: Triglyceride glucose
