# Supplementary figures and images for: Triglyceride glucose index is a useful marker for predicting subclinical coronary artery disease in the absence of traditional risk factors
Source: Lipids Health Dis. 2020 Jan 14;19:7. doi: 10.1186/s12944-020-1187-0 (PMC6961240; doi:10.1186/s12944-020-1187-0)

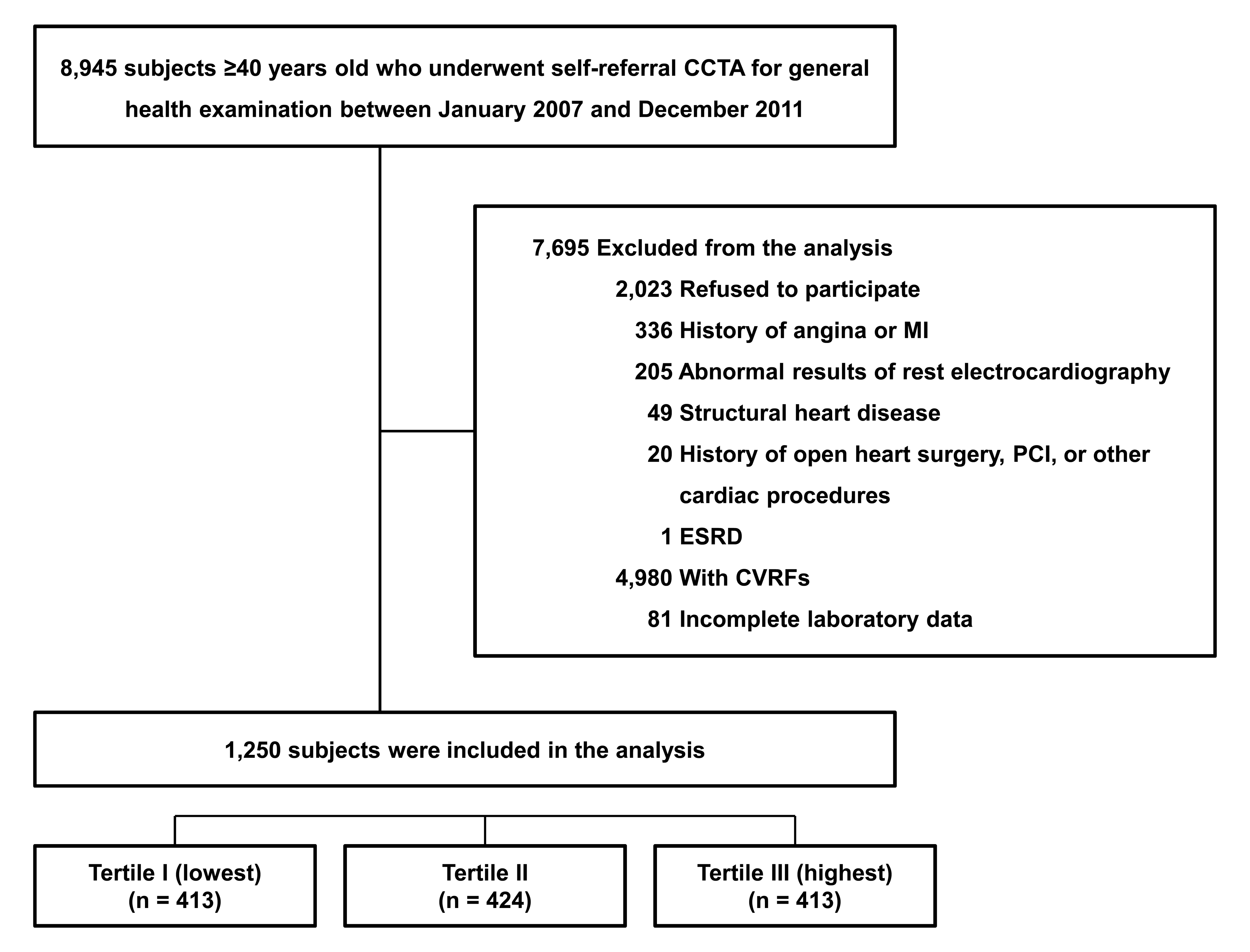

Supplement: Supplementary file 1 — Additional file 1: Figure S1. Overview of the study population. [file 12944_2020_1187_MOESM1_ESM.tif]

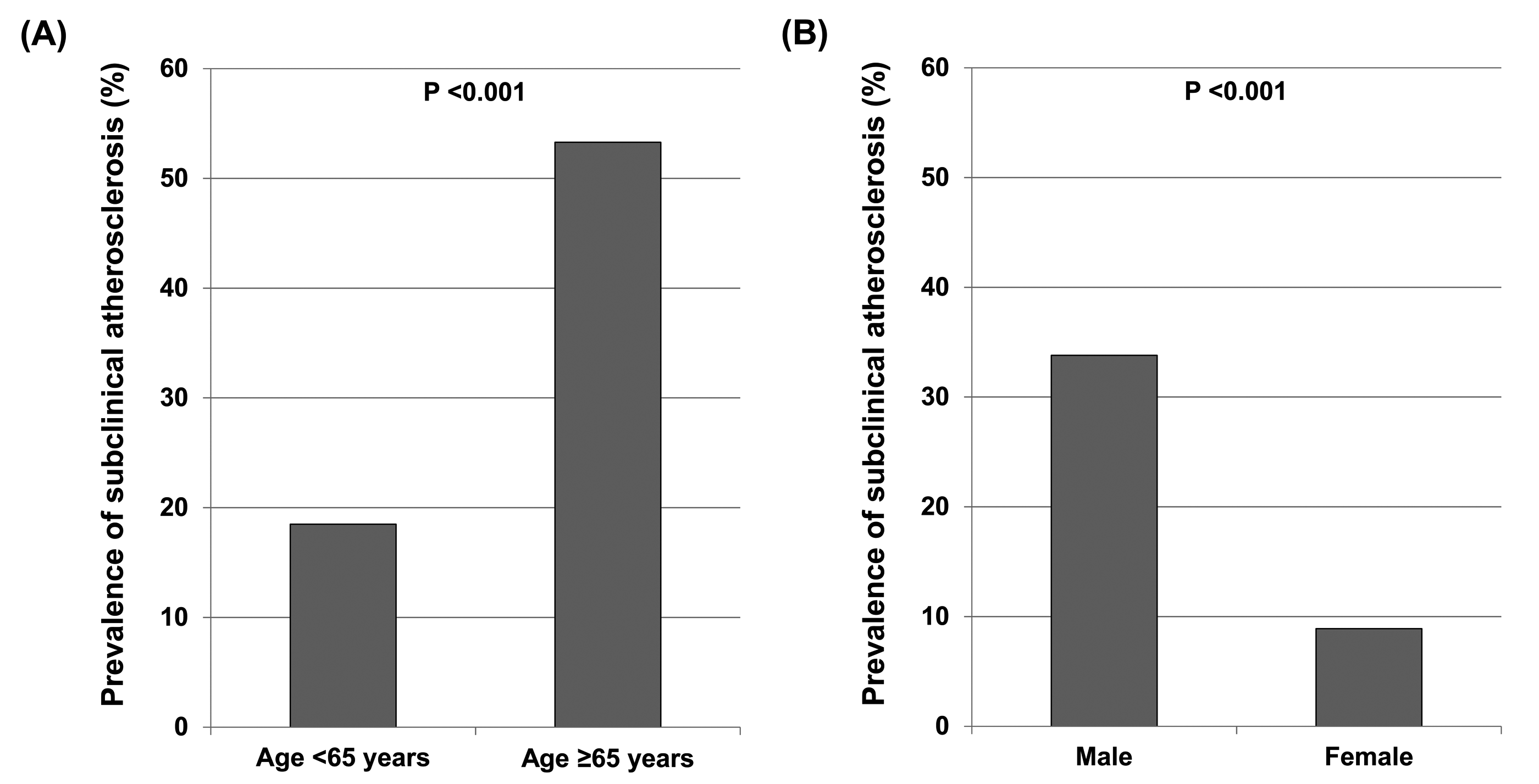

Supplement: Supplementary file 2 — Additional file 2: Figure S2. Prevalence of subclinical CAD according to old age and sex. [file 12944_2020_1187_MOESM2_ESM.tif]

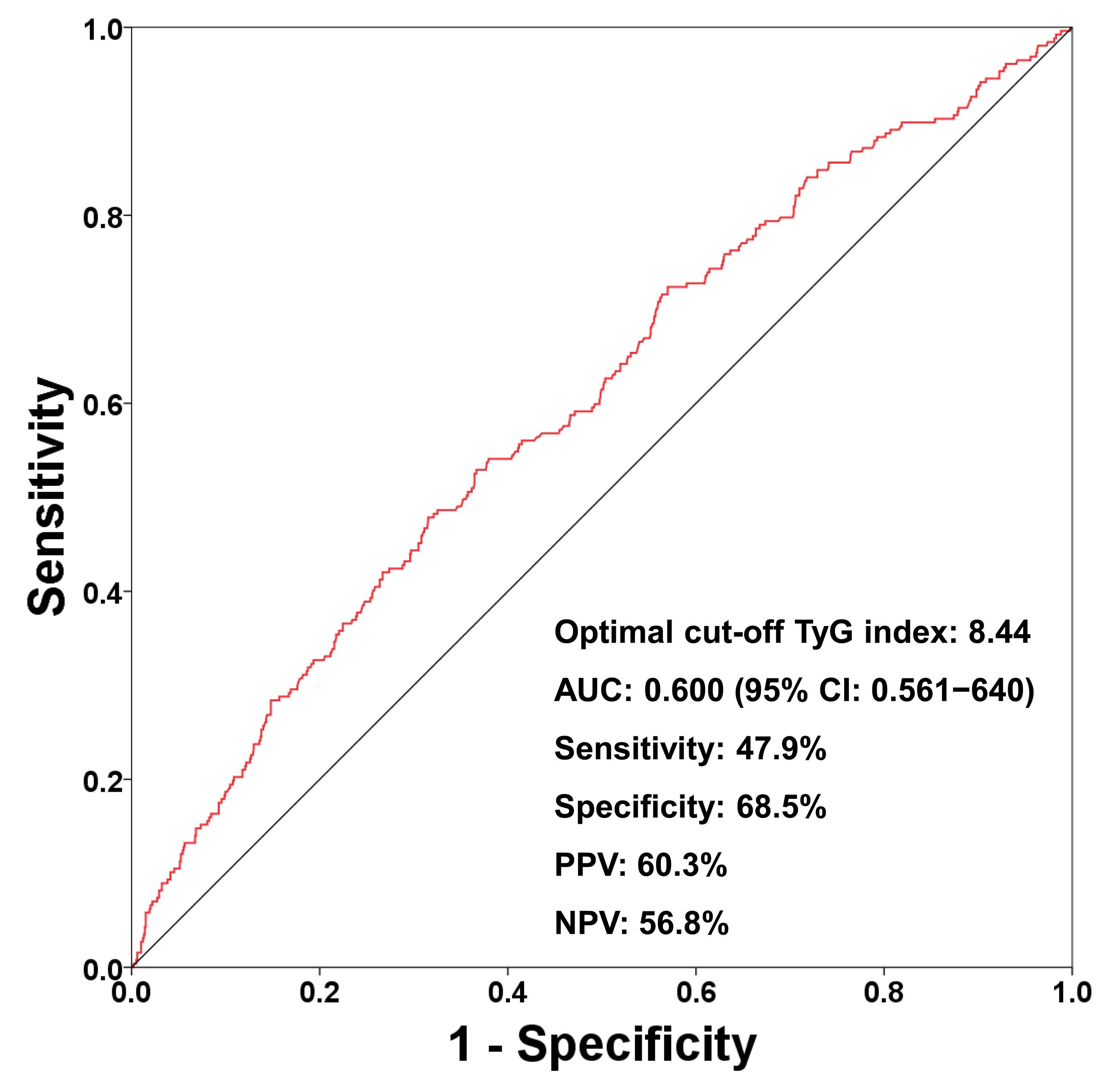

Supplement: Supplementary file 3 — Additional file 3: Figure S3. Optimal TyG cut-offs for predicting subclinical CAD. [file 12944_2020_1187_MOESM3_ESM.tif]
